# Awareness and Use of Nutrition Labels Among Adult Primary Healthcare Visitors in Taif, Saudi Arabia, in 2024: A Cross-Sectional Study

**DOI:** 10.7759/cureus.94962

**Published:** 2025-10-20

**Authors:** Adel Alamri, Hani Alaofi

**Affiliations:** 1 Family Medicine, Taif Health Cluster, Taif, SAU; 2 Family Medicine, Ministry of Health, Taif, SAU

**Keywords:** chronic diseases, consumer behavior, food choices, health awareness, nutrition labels, public education

## Abstract

Background

Nutrition labels are very important for helping people, especially those with long-term illnesses, make smart food choices as people become more health-conscious. However, a lot of labels are confusing, which leads to poor health outcomes associated with diet. This study aimed to determine how Taif's adult population understood and used nutritional value labels on pre-packaged foods.

Methodology

A cross-sectional study was conducted in 2024 across 18 primary healthcare centers (PHCCs) in Taif, Saudi Arabia, targeting a systematic random sample of adult residents (≥18 years) who attended the centers and provided consent. A self-administered questionnaire was used to collect data regarding participants' sociodemographic characteristics, and food and nutrition label awareness and use.

Results

A total of 404 adults in Taif were surveyed for the study; their mean age was 33.2±12.4 years, and 53% of them were women and 96% were Saudi citizens. Postgraduate participants were far more likely than primary-educated participants to observe food labels on pre-packaged foods (76.5% versus 25%, p=0.002). Compared to those who were single or divorced, married people exhibited more label awareness (70.3% versus 61.2% versus 0%, p<0.001). Those with more than six family members were more likely to notice labels than those with fewer than three (76.3% versus 58.1%, p=0.002). People with a family history of illnesses were also more aware (76.2% versus 58.3%, p=0.001), as were people with normal or overweight self-reported body mass indices (BMIs) as opposed to underweight and obese people (66.9% and 65.8% versus 50%, p=0.037). Lastly, individuals who were following a particular diet paid much more attention to nutrition labels than those who were not (86% versus 55.3%, p<0.001).

Conclusion

Many adults who attended Taif's PHCCs were aware of food labels, particularly those with higher levels of education, normal body mass indices, or special dietary needs. The most often used nutritional information was sugars, fats, and sodium, and they regularly verified production and expiration dates.

## Introduction

As most people nowadays are paying more attention to their dietary habits and are more aware of the significance of diet to optimize general health, their needs for nutritional information are also reasonably increasing [1]. Therefore, nutritional value labels on pre-packaged food are very useful, particularly for people who are on special diets, such as diabetic, hypertensive, dyslipidemic, obese, and cardiac patients who need to choose suitable foods according to their health status [2]. Furthermore, the World Health Organization (2003) has stated that nutrition and nutritional factors are responsible for almost 30% of cancers in the developed world [3].

Nutritional information on food labels helps people to be more aware of the nutritional value of different food products and enables them to compare different pre-packaged foods. This facilitates making healthy decisions when choosing a pre-packaged product, especially if there is more than one of the same category [4]. This behavior is very important, particularly during a period when there is an increasing prevalence of chronic diseases in the developing world, where the consumption of this type of food is also increasing. In this regard, Saudi and Gulf Cooperation Council (GCC) regulations follow Codex principles for food labeling [5].

Most imported and locally manufactured pre-packaged food products provide consumers with nutrition information that is usually added on food labels [1]. Most of the time, nutritional information on food labels is confusing and not easy to understand [6,7]. Moreover, there is a connection between insufficient understanding of nutrition labels and the high prevalence of chronic diseases, specifically those related to diet [1,8].

Sociodemographic characteristics of consumers are valuable predictors of the ability to understand and utilize nutrition labels [9-13].

A few studies have assessed consumers' awareness of nutritional values on food labels in the Gulf. From these attempts, despite consumers' acknowledgement of how important food labels are, they considered other information about production and expiration to be more important [2]. This study intends to investigate the level of awareness and usage of nutritional value labels among adults attending primary healthcare centers (PHCCs) in Taif, given the paucity of research on consumer awareness of these labels in Saudi Arabia and none specifically from Taif. It also aims to determine which nutritional elements are most frequently examined on pre-packaged food labels and investigate factors that affect awareness.

## Materials and methods

Study design, area, and population

A cross-sectional study design was employed. The study was conducted at PHCCs belonging to the Ministry of Health, Taif, Kingdom of Saudi Arabia. PHCCs in Saudi Arabia provide free of charge services of high quality. Thus, almost all people are following these centers, representing the general community adult population in Taif, which lies in the Makkah region in the western part of Saudi Arabia, with an area of about 13,840 km^2^. The Taif governorate's population is estimated at 993.8 thousand people in 2014, representing 12.79% of the total population of the region. Eighteen PHCCs provide primary care services to their visitors, as well as high-quality care in family and community medicine [14]. All adult men and women (18 years and above) who attended the PHCCs of the Ministry of Health in Taif during the study period (2021) and consented to participating in the study were eligible.

Sample size and selection criteria

By using Epi Info version 7 (Centers for Disease Control and Prevention, Atlanta, GA), the sample size was calculated as 374 adults based on a previous study carried out recently in Bahrain, with an awareness prevalence of 42%, a confidence interval (CI) of 95%, and acceptable margins of error (5%). The sample was increased by 10% to 404 to compensate for possible non-response. Visitors from other cities, as well as those who came for acute/emergency cases, were excluded.

Data collection, tool, and entry

The researcher chose five PHCCs using a simple random technique. The number of subjects was chosen daily by the systematic random sample technique according to the total number of visitors in each center/day. Approximately one month was needed to finish data collection. A questionnaire was used for data collection. It has been used previously in a study conducted in Ghana [12]. Permission to use the questionnaire was obtained from the author through email. The questionnaire includes four sections: sociodemographic characteristics (modified according to Saudi norms), health-related data, food and nutrition label awareness, and food and nutrition label use. The questionnaire was translated into Arabic, back-translated to English, and revised and validated by three experts (content validity). In addition, it was subjected to pilot testing on 20 volunteers and investigated for reliability and internal consistency using Cronbach's alpha test. A value of 0.73 has been obtained, which is acceptable.

Statistical analysis

The Statistical Package for the Social Sciences program version 26 (IBM Corp., Armonk, NY) was utilized for statistical analysis. The level of significance was determined using p-values (≤0.05). Descriptive statistics, such as frequency and percentage, were used for categorical variables, and mean ± standard deviation (SD) was used for continuous variables. Chi-square statistical test was utilized to test for the association and/or difference between two categorical variables. Fisher's exact test was applied instead of the Chi-square test in case of small frequencies. Student's t-test was used to compare the means of the continuous variable between two different groups. Multivariate logistic regression analysis, presented as adjusted odds ratio (aOR) and 95% confidence intervals (CIs), was performed to control for the confounding effect.

## Results

The study included 404 participants. Their sociodemographic characteristics are presented in Table 1. Women represented 53% of them. Their age ranged between 18 and 77 years (mean±SD: 33.2±12.4 years). Most of them (96%) were Saudi nationals, and 59.9% were university graduates. Around 51% of the participants were single, and 49.5% had family sizes ranging from four to six individuals. The number of children under 18 years in the household exceeded three in 11.9% of the participants, whereas 28.2% had no children under 18 years. Most of them (80.5%) were couples with children. Regarding employment status, 40.6% were employed, and 42.2% had an income of less than 5,000 SR (1,333 USD)/month.

**Table 1:** Sociodemographic characteristics of the participants (N=404) Categorical data are presented as frequencies and percentages, while numerical values are presented as mean and SD. Chi-square test SD: standard deviation, SR: Saudi riyal

Variables	Categories	Frequency	Percentage
Sex	Male	190	47
	Female	214	53
Nationality	Saudi	388	96
	Non-Saudi	16	4
Educational level	Primary school	16	4
	Intermediate school	40	9.9
	Secondary school	72	17.8
	University	242	59.9
	Postgraduate	34	8.4
Marital status	Single	206	51
	Married	182	45
	Divorced	16	4
Family size	≤3	86	21.3
	4-6	200	49.5
	>6	118	29.2
Number of household children <18 years	No	114	28.2
	1	110	27.2
	2	74	18.3
	3	58	14.4
	>3	48	11.9
Household composition (n=398)	Single parent with children	40	10
	A couple with children	320	80.5
	A couple with no children	38	9.5
Employment status	Unemployed	100	24.8
	Employed	164	40.6
	Student	108	26.7
	Retired	32	7.9
Average monthly income (SR/month)	≤5,000	170	42.2
	5,001-8,000	62	15.3
	8,001-12,000	62	15.3
	12,001-15,000	64	15.8
	>15,000	46	11.4

As shown in Table 2, medical and health problems were reported by most of the participants (83.7%). The most frequent were dental problems (28.2%), food allergy/intolerance (21.3%), and diabetes (11.4%). A family history of medical diseases was reported by almost one-third of the participants (31.2%). Among them (n=126), diabetes was the most common (69%).

**Table 2:** History of medical problems among the participants

History of medical problems	Number (%)
No	66 (16.3)
Food allergy/intolerance	86 (21.3)
Diabetes	46 (11.4)
Cancer	2 (0.5)
Hypertension	40 (9.9)
High cholesterol	20 (5.0)
Dental problems	114 (28.2)
Obesity	34 (8.4)
Gout	18 (4.5)
Kidney disease	6 (1.5)
Gastric/duodenal ulcer	20 (5.0)
Others	62 (15.3)

A familial history of food allergy/intolerance was found in 6.3% of the participants, as shown in Table 3. 

**Table 3:** Family history of medical problems among the participants (n=126) Data are presented as numbers and percentages.

Family history of medical problems	Number (%)
Food allergy/intolerance	8 (6.3)
Diabetes	87 (69.0)
Cancer	4 (3.2)
Hypertension	45 (9.9)
High cholesterol	5 (5.0)
Obesity	3 (2.4)
Kidney disease	2 (1.6)
Others	2 (1.6)

BMI

Approximately 18.8% of the subjects were overweight, whereas 12.9% were obese.

History of being on a special diet

Almost one-quarter (24.8%) of the participants were on a special diet prescribed by a doctor or nutritionist/dietician for a health condition.

Food and nutritional label awareness

**Figure 1:**
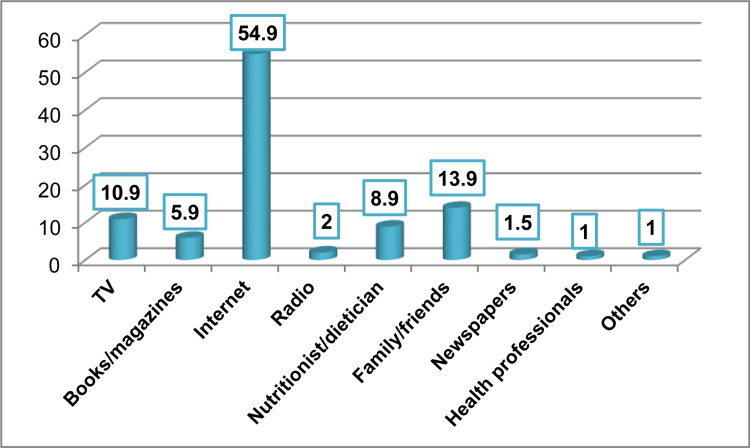
Main sources of information about food and nutritional awareness among the participants

As demonstrated in Figure 1, the main source of information about food and nutritional awareness among the participants was the Internet (54.9%), followed by family and friends (13.9%), television (10.9%), and a nutritionist/dietician (8.9%).

Around 67.3% of the participants were aware that some pre-packaged foods carry a food label, and 62.9% had noticed/seen the food label on any pre-packaged food. Also, 64.9% of the participants were aware that some food labels contain nutrition information about the food, and 58.4% had noticed/seen the nutrition information on any pre-packaged food. Less than half of the participants (45.6%) rated their level of understanding of the nutrition information provided on food labels as moderate, while 35.6% rated it as high, as shown in Table 4.

**Table 4:** Assessment of awareness of the participants regarding food labels on pre-packaged foods Categorical variables are reported as frequencies and percentages.

Food labels on pre-packaged foods	Frequency	Percentage
Are you aware that some pre-packaged foods carry a food label?		
No	132	32.7
Yes	272	67.3
Have you ever noticed/seen the food label on any pre-packaged food?		
No	150	37.1
Yes	254	62.9
Are you aware that some food labels contain nutrition information about the food?		
No	142	35.1
Yes	262	64.9
Have you ever noticed/seen the nutrition information on any pre-packaged food?		
No	168	41.6
Yes	236	58.4
How would you rate your level of understanding of the nutrition information provided on food labels?		
Low	76	18.8
Moderate	184	45.6
High	144	35.6

In Table 5, it is shown that participants with higher educational attainment (postgraduates) were more likely to notice the food label on pre-packaged foods compared to those who were less educated, particularly, primary school educated participants (76.5% versus 25%, p=0.002). Also, 70.3% of the married participants and 61.2% of the single participants, compared to none of the divorced participants, noticed the food labels on pre-packaged foods (p<0.001). Most of the participants (76.3%) whose family size exceeded six, compared to 58.1% of those whose family size was three or less, noticed the food label on any pre-packaged foods (p=0.002). Most of the participants (76.2%) with a family history of medical diseases, compared to 58.3% of those without such a history, noticed the food labels on pre-packaged foods (p=0.001). Regarding the participants' BMI, normal (66.9%) and overweight (65.8%) participants were more likely to notice the food label on any pre-packaged food compared to underweight and obese (50%, p=0.037). Most of the participants on a special diet (86%), compared to 55.3% of those not on a special diet, reported noticing the food labels on pre-packaged foods (p<0.001), as shown in Table 6.

**Table 5:** Factors associated with seeing/noticing the food label on any pre-packaged food The Chi-square statistical test was used to test for the association and/or difference between two categorical variables. Fisher's exact test was applied instead of the Chi-square test in cases of small frequencies. Student's t-test was used to compare the means of the continuous variable between two different groups. A p-value is considered statistically significant if it is below a predetermined threshold, typically p<0.05 (5% significance level). *Fisher's exact test, ⱶStudent's t-test, **Chi-square test OR: odds ratio, CI: confidence interval, SD: standard deviation, SR: Saudi riyal

Factors	Seeing/noticing the food label on any pre-packaged food (number (%))		Crude OR (95% CI)	Chi-square/t value	p-value
	No (N=150)	Yes (N=254)			
						Sex					
						Male (n=190)	72 (37.9)	118 (62.1)	1	0.09	0.764**
Female (n=214)	78 (36.4)	136 (63.6)	1.06 (0.71-1.59)								
Age (years)					
Mean±SD	34.1±14.4	32.7±11.0	0.99 (0.98-1.01)	1.071	0.285^ⱶ^
Nationality					
Saudi (n=388)	146 (37.6)	242 (62.4)	1	NA	0.227*
Non-Saudi (n=16)	4 (25.0)	12 (75.0)	1.81 (0.57-5.72)		
Educational level					
Primary school (n=16)	12 (75.0)	4 (25.0)	1	16.55	0.002**
Intermediate school (n=40)	20 (50.0)	20 (50.0)	3.00 (0.83-10.90)		
Secondary school (n=72)	28 (38.9)	44 (61.1)	4.71 (1.38-10.08)		
University (n=242)	82 (33.9)	160 (66.1)	5.85 (1.83-18.72)		
Postgraduate (n=34)	8 (23.5)	26 (76.5)	9.75 (2.45-38.81)		
Marital status					
Single (n=206)	80 (38.8)	126 (61.2)	1	31.687	<0.001**
Married (n=182)	54 (29.7)	128 (70.3)	1.50 (0.99-2.30)		
Divorced (n=16)	16 (100)	0 (0.0)	0.02 (0.001-0.33)		
Family size					
≤3 (n=86)	36 (41.9)	50 (58.1)	1	12.855	0.002**
4-6 (n=200)	86 (43.0)	114 (57.0)	0.95 (0.57-1.59)		
>6 (n=118)	28 (23.7)	90 (76.3)	2.37 (1.30-4.32)		
Number of household children <18 years					
No (n=114)	44 (38.6)	70 (61.4)	1	3.551	0.470**
1 (n=110)	44 (40.0)	66 (60.0)	0.94 (0.55-1.61)		
2 (n=74)	28 (37.8)	46 (62.2)	1.03 (0.57-1.89)		
3 (n=58)	22 (37.9)	36 (62.1)	1.03 (0.54-1.97)		
>3 (n=48)	12 (25.0)	36 (75.0)	1.89 (0.89-4.01)		
Household composition (n=398)					
Single parent with children (n=40)	16 (40.0)	24 (60.0)	1	0.602	0.740**
Couple with children (n=320)	116 (36.3)	204 (63.7)	1.17 (0.60-2.30)		
Couple with no children (n=38)	12 (31.6)	26 (68.4)	1.44 (0.57-3.67)		
Employment status					
Unemployed (n=100)	36 (36.0)	64 (64.0)	1	1.017	0.797**
Employed (n=164)	58 (35.4)	106 (64.6)	1.03 (0.61-1.73)		
Student (n=108)	42 (38.9)	66 (61.1)	0.88 (0.50-1.55)		
Retired (n=32)	14 (43.8)	18 (56.2)	0.72 (0.32-1.62)		
Average monthly income (SR/month)					
≤5,000 (n=170)	72 (42.4)	98 (57.6)	1	3.746	0.442**
5,001-8,000 (n=62)	20 (32.3)	42 (67.7)	1.54 (0.84-2.85)		
8,001-12,000 (n=62)	22 (35.5)	40 (64.5)	1.34 (0.73-2.44)		
12,001-15,000 (n=64)	20 (31.2)	44 (68.8)	1.62 (0.88-2.97)		
>15,000 (n=46)	16 (34.8)	30 (65.2)	1.38 (0.70-2.72)		
History of medical problems					
No (n=66)	18 (27.3)	48 (72.7)	1		
Yes (n=338)	132 (39.1)	206 (60.9)	0.59 (0.33-1.05)	3.283	0.070**
Family history of medical diseases					
No (n=230)	96 (41.7)	134 (58.3)	1	15.077	0.001**
Yes (n=126)	30 (23.8)	96 (76.2)	2.29 (1.41-3.73)		
Don't know (n=48)	24 (50.0)	24 (50.0)	0.72 (0.38-1.34)		
Body mass index of the participants					
Underweight (n=40)	20 (50.0)	20 (50.0)	1	8.488	0.037**
Normal (n=236)	78 (33.1)	158 (66.9)	2.03 (1.03-3.98)		
Overweight (n=76)	26 (34.2)	50 (65.8)	1.92 (0.88-4.20)		
Obese (n=52)	26 (50.0)	26 (50.0)	1.00 (0.44-2.28)		
History of being on a special diet					
No (n=304)	136 (44.7)	168 (55.3)	1	30.454	<0.001**
Yes (n=100)	14 (14.0)	86 (86.0)	4.97 (2.71-9.14)		

**Table 6:** Predictors of seeing/noticing the food label on any pre-packaged food among the participants: multivariate logistic regression analysis For each categorical variable, the reference category is indicated by the superscript "a". The table includes the B, SE, aOR, 95% CI, and p-value for each level of the included variables. Terms of family size and body mass index were insignificant and removed from the final model. a: reference category, B: slope, SE: standard error, aOR: adjusted odds ratio, CI: confidence interval

	B	SE	aOR	95% CI	p-value
Educational level					
Primary school^a^					
Intermediate school	0.877	0.702	2.4	0.61-9.52	0.211
Secondary school	1.566	0.676	4.79	1.27-18.01	0.021
University	2.041	0.65	7.7	2.15-27.52	0.002
Postgraduate	1.698	0.771	5.46	1.21-24.76	0.028
Marital status					
Single^a^					
Married	0.702	0.27	2.02	1.19-3.43	0.009
Divorced	-3.016	0.972	0.21	0.09-0.63	<0.001
History of being on a special diet					
No^a^					
Yes	1.366	0.33	3.92	2.05-7.48	<0.001
Family history of medical diseases					
No^a^					
Yes	0.598	0.278	1.82	1.05-3.14	0.031
Don't know	-0.007	0.357	0.99	0.49-2.00	0.985

Food and nutrition label use

History of reading food labels on pre-packaged foods "most of the time" was reported by 19.9%, while history of reading food labels only "sometimes" was reported by 71.2% of the participants who were aware that some pre-packaged foods carry a food label and/or that some food labels contain nutrition information about the food.

Participants who have a history of reading food labels were most likely to look out for the expiratory date (79.3%), the manufacture date (66.9%), brand name (60.2%), and country of origin (56%), as shown in Table 7.

**Table 7:** Factors associated with seeing the nutrition information on any pre-packaged food Categorical variables are reported as frequencies and percentages. *Fisher's exact test, ⱶStudent's t-test, **Chi-square test OR: Odds ratio, CI: Confidence interval, SD: standard deviation, SR: Saudi riyal

Factors	Seeing/noticing the nutrition information on any pre-packaged food (number (%))		Crude OR (95% CI)	Chi-square/ t value	p-value
	No (N=168)	Yes (N=236)			
						Sex					
						Male (n=190)	84 (44.2)	106 (55.8)	1	1.019	0.313**
Female (n=214)	84 (39.3)	130 (60.7)	1.23 (0.82-1.82)								
Age (years)					
Mean±SD	34.4±14.2	32.4±10.9	0.99 (0.97-1.0)	1.625	0.105^ⱶ^
Nationality					
Saudi (n=388)	164 (42.3)	224 (57.7)	1	NA	0.132*
Non-Saudi (n=16)	4 (25.0)	12 (75.0)	2.20 (0.70-6.93)		
Educational level					
Primary school (n=16)	12 (75.0)	4 (25.0)	1	15.246	0.004*
Intermediate school (n=40)	22 (55.0)	18 (45.0)	2.45 (0.67-8.93)		
Secondary school (n=72)	30 (41.7)	42 (58.3)	4.20 (1.23-14.29)		
University (n=242)	96 (39.7)	146 (60.3)	4.56 (1.43-14.56)		
Postgraduate (n=34)	8 (23.5)	26 (76.5)	9.75 (2.45-38.81)		
Marital status					
Single (n=206)	92 (44.7)	114 (55.3)	1	28.842	<0.001*
Married (n=182)	60 (33.0)	122 (67.0)	1.64 (1.09-2.48)		
Divorced (n=16)	16 (100)	0 (0.0)	0.02 (0.001-0.41)		
Family size					
≤3 (n=86)	36 (41.9)	50 (58.1)	1	6.692	0.035**
4-6 (n=200)	94 (47.0)	106 (53.0)	0.81 (0.49-1.35)		
>6 (n=118)	38 (32.2)	80 (67.8)	1.52 (0.85-2.70)		
Number of household children <18 years					
No (n=114)	52 (45.0)	62 (54.4)	1	4.839	0.304**
1 (n=110)	50 (45.5)	60 (54.5)	1.01 (0.59-1.70)		
2 (n=74)	30 (40.5)	44 (59.5)	1.23 (0.68-2.22)		
3 (n=58)	22 (37.9)	36 (62.1)	1.37 (0.72-2.62)		
>3 (n=48)	14 (29.2)	34 (70.8)	2.04 (0.99-4.19)		
Household composition (n=398)					
Single parent with children (n=40)	20 (50.0)	20 (50.0)	1	1.733	0.420**
Couple with children (n=320)	128 (40.0)	192 (60.0)	1.50 (0.78-2.90)		
Couple with no children (n=38)	14 (36.8)	24 (63.2)	1.71 (0.69-4.24)		
Employment status					
Unemployed (n=100)	36 (36.0)	64 (64.0)	1	2.581	0.461**
Employed (n=164)	68 (41.5)	96 (58.5)	0.79 (0.48-1.33)		
Student (n=108)	48 (44.4)	60 (55.6)	0.70 (0.40-1.23)		
Retired (n=32)	16 (50.0)	16 (50.0)	0.56 (0.25-1.26)		
Average monthly income (SR/month)					
≤5,000 (n=170)	76 (44.7)	94 (55.3)	1	6.915	0.140**
5,001-8,000 (n=62)	24 (38.7)	38 (61.3)	1.28 (0.71-2.32)		
8,001-12,000 (n=62)	30 (48.4)	32 (51.6)	0.86 (0.48-1.54)		
12,001-15,000 (n=64)	18 (28.1)	46 (71.9)	2.07 (1.11-3.85)		
>15,000 (n=46)	20 (43.5)	26 (56.5)	1.05 (0.55-2.03)		
History of medical problems					
No (n=66)	26 (39.4)	40 (60.6)	1	0.156	0.693**
Yes (n=338)	142 (42.0)	196 (58.0)	0.90 (0.52-1.54)		
Family history of medical diseases					
No (n=230)	112 (48.7)	118 (51.3)	1	16.202	<0.001**
Yes (n=126)	34 (27.0)	92 (73.0)	2.57 (1.60-4.11)		
Don't know (n=48)	22 (45.8)	26 (54.2)	1.12 (0.60-2.09)		
Body mass index of the participants					
Underweight (n=40)	24 (60.0)	16 (40.0)	1	8.96	0.030**
Normal (n=236)	90 (38.1)	146 (61.9)	2.43 (1.23-4.83)		
Overweight (n=75)	28 (36.8)	48 (63.2)	2.57 (1.17-5.64)		
Obese (n=52)	26 (50.0)	26 (50.0)	1.50 (0.65-3.45)		
History of being on a special diet					
No (n=304)	150 (49.3)	154 (50.7)	1	30.429	<0.001**
Yes (n=100)	18 (18.0)	82 (82.0)	4.44 (2.54-7.75)		

A history of using nutrition information on food labels on the pre-packaged foods "most of the time" was reported by 19.9%, while a history of reading food labels only "sometimes" was reported by 62.8% of the participants who reported that they read food labels on pre-packaged foods.

These participants were most likely to look out for sugars (79.1%), fats (71.4%), carbohydrates (59.1%), and sodium/salts (57.3%), as illustrated in Table 8.

**Table 8:** Predictors of seeing the nutrition information on any pre-packaged food among the participants: multivariate logistic regression analysis For each categorical variable, the reference category is indicated by the superscript "a". The table includes the B, SE, aOR, 95% CI, and p-value for each level of the included variables. Terms of family size and body mass index were insignificant and removed from the final model. a: reference category, B: slope, SE: standard error, aOR: adjusted odds ratio, CI: confidence interval

Factor	B	SE	aOR	95% CI	p-value
Educational level					
Primary school^a^					
Intermediate school	0.642	0.698	1.9	0.48-7.46	0.358
Secondary school	1.566	0.674	4.87	1.30-18.27	0.019
University	1.806	0.643	6.08	1.73-21.46	0.005
Postgraduate	1.645	0.765	5.18	1.16-23.21	0.031
Marital status					
Single^a^					
Married	0.664	0.26	1.94	1.17-3.23	0.011
Divorced	-3.119	0.908	0.22	0.11-0.69	<0.001
History of being on a special diet					
No^a^					
Yes	1.325	0.304	3.76	2.07-6.83	<0.001
Family history of medical diseases					
No^a^					
Yes	0.773	0.264	2.17	1.28-3.66	0.004
Don't know	0.498	0.357	1.65	0.82-3.31	0.164

Ways through which reading nutrition labels helps the participants

**Figure 2:**
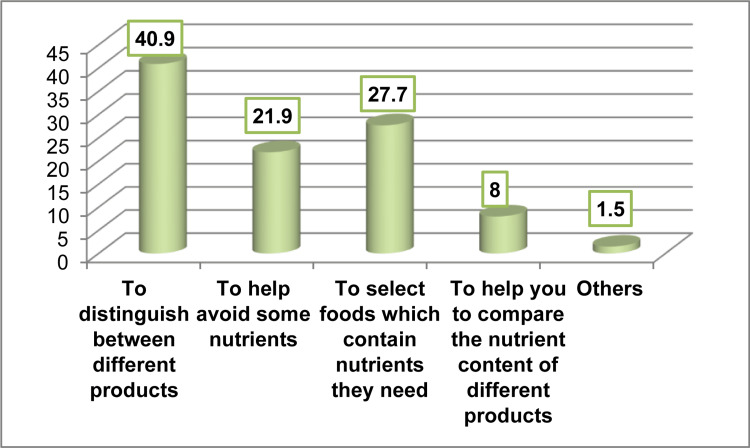
Ways through which reading nutrition labels helps the participants when they are deciding on what pre-packaged foods to buy (n=220)

Figure 2 shows that reading nutrition labels helped participants to distinguish between different products (40.9%), select foods that contained nutrients they needed (27.7%), and avoid some nutrients (21.9%).

Circumstances when participants usually use the nutrition information provided on pre-packaged foods

**Figure 3:**
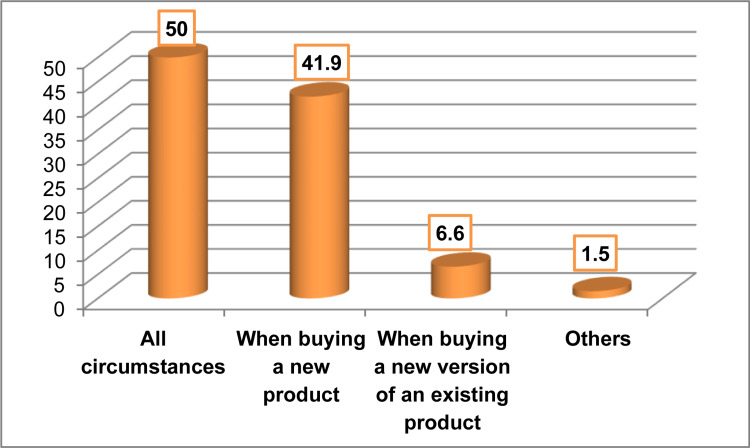
Circumstances in which participants usually use the nutrition information provided on pre-packaged foods (n=220)

Regarding the circumstances when participants usually use the nutrition information provided on pre-packaged foods, half of the participants reported doing so always, whereas 41.9% reported doing so only when buying a new product, as displayed in Figure 3.

## Discussion

Noticing and reading the nutritional food information is considered an effective strategy to make healthy and informed food choices, prevent obesity, and control hypertension [5]. In line with that, the present study was carried out to explore the awareness of the adult population about nutritional value labels on pre-packaged food and the pattern of their utilization in Taif, Kingdom of Saudi Arabia.

Studies carried out regionally and internationally revealed different findings regarding awareness and utilization of nutritional labels on pre-packaged foods. In Bahrain, only 42% of the consumers read the food label; in the United Arab Emirates, awareness of consumers about reading the food label was very high (89.5%) [1,2]. In Ghana, 98.4% had an idea of what food labels were; however, only 66.7% understood them. Although the majority looked at food labels, only 51.9% claimed to do so "always" [11]. In Nigeria, only 5% of adult consumers were adequately aware of food labels [12]. In an Indian study, 92.2% of the participants were aware of food labels on pre-packed food items, and 99.3% preferred buying pre-packaged food with labels; however, only 76% checked for food labels "every time," and only 7.2% found it simple to do so [14]. In Malaysia, only 21.6% of the students "often" used the food label when making food purchasing decisions [15]. The apparent differences between various studies in this regard should be interpreted in terms of the differences in the demographic characteristics of the participants, as well as the use of different measurement tools by the studies.

Similar to what has been observed in the current study, consumers in Bahrain and the United Arab Emirates mainly read basic information such as manufacture and expiry dates. In Ghana, 89.3% of the consumers were affected by "key factors" on food labels, with nutritional content and date of expiration being the factors that the participants paid most attention to [1,2]. However, at the point of purchase, 79.4% looked out for the expiry date [11]. In Malawi, price was the major determinant of purchase, while in Lesotho, the major determinant was nutrition information itself [15,16]. In Zimbabwe, consumers only considered brand, nutritional profile, and mass of a product concerning the price, and they seemed not to understand some of the information on the food labels [17]. In India, the date of production and expiration appeared to be the most checked items (86%) [18]. In another Indian study, the items checked frequently were price, instructions for use, ingredients, production, and expiry date [14]. In Malaysia, important aspects while buying food products were expiry date (98.5%), taste (95.7%), price (92.4%), and nutrient content (90.5%) [19]. Again, variations between different studies, including the present one, are affected mainly by the sociodemographic characteristics of the participants, as well as their cultural backgrounds.

Similar to what has been reported in a Bahraini study, most of the consumers in the present study reported reading mainly about the amount of sugars and fats while buying the food products. This may be attributed to the high prevalence of diabetes mellitus and hypertension in this region [2].

In the current study, heterogenecity was observed regarding the impact of educational level, and consumers who were more educated, married or single, had a large family size, had a family history of diseases, were of a normal BMI or overweight, and were on a special diet were more likely to notice the food labels and see the nutrition information on pre-packaged foods than others. In Ghana, the level of understanding labels was highly related to gender, age, occupation, educational level, and income of the consumers. The most important factor was gender in the multivariate analysis. Additionally, label utilization was highly related to age, occupation, and educational level, with the latter being the most important factor in the multivariate analysis [11]. In Nigeria, consumers' age was significantly associated with the level of knowledge of food label information, as the level of knowledge increased with increasing age. Also, there was a clear relation between food label usage and respondents' income [12]. In India, reading labels was significantly associated with the level of education, socioeconomic status, and consumers' weight [18].

The impact of consumers' gender on awareness and utilization of nutrition information on pre-packaged foods is unclear. However, most studies have reported that women are more likely to utilize nutrition information provided on pre-packaged foods than men [20,21]. According to them, this is mainly attributed to the role that women play in the process of food selection [22]. On the other hand, relatively fewer studies found that men expressed higher usage and perception of the importance of the nutrition label compared to women. In the present survey, consumers' gender had no role in the awareness and utilization of nutritional information of pre-packaged foods [11,23].

Strengths and limitations

This study is unique and is important for discovering the ways through which reading nutrition labels helps consumers decide what pre-packaged foods to buy. However, it has some limitations. First, the temporal relationship between exposure and outcome cannot be determined because of the study's cross-sectional nature. Second, conducting the study only among attendees of PHCCs does not reflect the actual situation of the whole community. However, they were the most accessible group. Also, there is a limitation as regards the imbalance in sample size between studied groups in some variables, such as educational level, which may reduce statistical power in the smaller groups and affect the robustness between group comparisons.

## Conclusions

A considerable proportion of adult PHCC attendees were aware that some pre-packaged foods carry a food label and nutrition information. Special groups (e.g., more educated single subjects, subjects with normal BMI, and those on a special diet) were more likely to notice the food label and see the nutrition information than others. Expiry and manufacturing dates were more likely to be looked for than other information. The history of using nutrition information on food labels on pre-packaged foods was reported by most of the participants who reported reading the food labels. The information most likely to be used by these participants pertained to sugars, fats, carbohydrates, and sodium/salts. The most frequently reported ways through which reading nutrition labels helps participants were to distinguish between different products, select foods that contain nutrients they need, and help avoid certain nutrients. The significance of reading nutritional labels and urging consumers to prioritize important health indicators over price or package features should be the focus of educational programs in public areas and media channels to raise public knowledge and promote informed food choices. Further studies across a range of demographics and more precise labeling are also necessary to better understand and encourage nutrition-conscious behavior.

## Appendices

Table 9 presents the questionnaires on the participants' sociodemographic data, Table 10 on medical history, Table 11 on food and nutritional label awareness, and Table 12 on food and nutrition label use. 

**Table 9:** Sociodemographic data

Sociodemographic data		
	Sex	☐Male ☐Female
	How old are you?	…………………..
	What is your nationality?	☐Saudi ☐ Non-Saudi
	What is your level of formal education?	☐Primary school ☐Intermediate school ☐Secondary school ☐University ☐Postgraduate
	What is your marital status?	☐Single ☐ Married ☐ Divorced
	How many people are in your household?	…………………..
	How many of the people in your household are children less than 18 years?	…………………..
	What is your household composition?	☐Single parent with children ☐A couple with no children ☐A couple with four children ☐Other (specify)…………..
	What is your employment status?	☐Unemployed ☐Employed (employee) ☐Student ☐ Retired
	What is your average monthly income (SR) (from all sources)?	☐≤5,000 ☐ 5,001-8,000 ☐8,001-12,000 ☐12,001-15,000 ☐ >15,000

**Table 10:** Medical history

A: Medical history		
Do you have any of the following health conditions (tick all that apply)?	Yes	No
Food allergy/intolerance		
Diabetes		
Cancer		
Hypertension		
High cholesterol		
Dental problems		
Obesity		
Gout		
Kidney disease		
Gastric/duodenal ulcer		
Others (specify)		
Do you have a family history of any of the above health conditions?	☐Yes ☐No ☐ Don't know If yes, state …………………………….	
What is your weight?		
What is your height?		
Are you on a special diet prescribed by a doctor or nutritionist/dietitian for a health condition?	☐Yes ☐No	

**Table 11:** Food and nutritional label awareness

B: Food and nutritional label awareness	
What is your main source of nutrition information (tick only one response)?	☐Television ☐Books and magazines ☐Internet ☐Radio ☐Nutritionist/dietician ☐Family and friends ☐Newspapers ☐Health professionals ☐Others (specify) ……………………………
Are you aware that some pre-packaged foods carry a food label?	☐Yes ☐No
Have you ever noticed/seen the food label on any pre-packaged food?	☐Yes ☐No
Are you aware that some food labels contain nutrition information about the food?	☐Yes ☐No
Have you ever noticed/seen the nutrition information on any pre-packaged food?	☐Yes ☐No
How would you rate your level of understanding of the nutrition information provided on food Labels?	☐High ☐Moderate ☐Low
If you answered "Yes" to any of the questions 17-20, then complete section C. If you answered "No" to questions 17-20, skip section C.	

**Table 12:** Food and nutrition label use

C: Food and nutrition label use	
Do you ever read food labels on pre-packaged foods that you buy?	☐No, never ☐Yes, sometimes ☐ Yes, often
If you answered "sometimes" or "often" to Q-22, what information are you most likely to look out for when you read/look at a food label? (Tick all that apply.)	☐Country of origin ☐Brand name ☐Manufacture date ☐Expiry date ☐Additives ☐Nutrition/health claims ☐Description of the food ☐Nutrition information ☐List of ingredients ☐Others (specify)………………………….
Do you ever use nutrition information (total calories, carbohydrate, protein, etc.) on food labels on the pre-packaged foods that you buy?	☐No, never ☐Yes, sometimes ☐ Yes, often
What information are you most likely to use when you read or look at a nutrition label? (Tick all that apply.)	☐Total energy (total calories) ☐Carbohydrate ☐Protein ☐Fats ☐Vitamins and minerals ☐Cholesterol ☐Fiber ☐Sugars ☐Sodium/salt ☐Others (specify)………………………….
How does reading nutrition labels help you when deciding on what pre-packaged foods to buy? (Tick only one response, please.)	☐To distinguish between different products ☐To help avoid some nutrients ☐To select foods that contain nutrients, you need to know ☐To help you compare the nutrient content of different products ☐Others (specify)……………………………
Under what circumstances do you usually use the nutrition information provided on pre-packaged foods? (Tick only one response, please.)	☐Under all circumstances ☐When buying a new product ☐When buying a new version of an existing product ☐Others (specify)………………………
